# Family Functioning in the Time of COVID-19 Among Economically Vulnerable Families: Risks and Protective Factors

**DOI:** 10.3389/fpsyg.2021.730447

**Published:** 2021-10-06

**Authors:** Minxuan He, Natasha Cabrera, Jone Renteria, Yu Chen, Angelica Alonso, S. Alexa McDorman, Marina A. Kerlow, Stephanie M. Reich

**Affiliations:** ^1^Department of Human Development and Quantitative Methodology, University of Maryland, College Park, College Park, MD, United States; ^2^School of Education, University of California, Irvine, Irvine, CA, United States

**Keywords:** COVID-19, parental mental health, parent engagement, socioemotional problems, prosocial behaviors, positivity, coparenting support

## Abstract

The ongoing COVID-19 crisis has been particularly harmful to economically vulnerable families with young children. We surveyed 247 low-income mothers and fathers from 142 families in the United States about changes in their family life following the economic and social restrictions imposed by the pandemic. We examined the associations between pandemic-related risk factors such as economic stressors (e.g., loss of job) and social stressors (e.g., exposure to the virus) on family functioning (e.g., parents’ mental health, parent engagement, and children’s socioemotional behaviors) and the degree to which coparenting support and parents’ positivity protected families from the negative effects of these stressors on their wellbeing. We found both positive and negative associations. Mothers and fathers who reported more economic stressors since the pandemic also observed that their children behaved more prosocially and that fathers experienced more mental health difficulties during the pandemic. Mothers and fathers who reported more social stressors reported that they were less engaged with their children and their children exhibited more behavior problems compared to before the pandemic. We also found that mothers and fathers who reported feeling more positive also reported feeling less depressed and stressed during the pandemic and observed that their children had more prosocial behaviors compared to before the pandemic. Compared to before the pandemic, mothers and fathers who reported a more supportive coparenting relationship also reported more parent engagement and observed more prosocial behaviors in their children. In terms of protective factors, high levels of parent positivity during the pandemic protected mothers (less mental health difficulties) whereas high levels of coparenting support protected fathers (less mental health difficulties) from the negative effects of economic stress on their mental health during the pandemic. These findings highlight family processes that could promote resilience in mothers and fathers in the face of pandemic-related economic and social stressors.

## Introduction

The ongoing COVID-19 crisis has disrupted all aspects of our lives resulting in unprecedented levels of social and economic distress. The social distancing, isolation, and country-wide lockdown measures to help reduce virus transmission, have also created stressful experiences for families and children. Individuals through their own behaviors and characteristics impact the functioning of the family as a group as well as the functioning of each individual within the family (Cox and Paley, 1997). Research on the effects of the COVID-19 pandemic, other pandemics, and natural disasters on factors that impact family functioning (hereby family functioning) suggests that there may be both immediate and long-term adverse consequences for many children, with early childhood being a particular risk factor (Magson et al., 2021). Studies conducted during the current pandemic show that many parents are facing unemployment; front-line essential jobs; working from home while caring for children; schooling children at home; dealing with economic uncertainty; and, managing a host of family stressors (Canady, 2020; Fontanesi et al., 2020). At the same time, children’s lives have also suddenly changed. During the current pandemic, children’s routines and childcare experiences have been drastically altered and many may find themselves at home with stressed adults and upset routines (Pachter et al., 2020; de Figueiredo et al., 2021). Overall, these stressors could take a toll on children’s ability to cope and on parents’ ability to manage the added stress. These stresses can dysregulate children and diminish parents’ ability to provide consistent care and support for their children, which can undermine the parent–child relationship and children’s socioemotional functioning.

A group that has been particularly affected by the COVID-19 pandemic is economically vulnerable families (Pew Research Center, 2020). Approximately 63% of young Black children and 57% of young Hispanic children ages 5 or younger live in low-income families, defined by incomes about two times the federal poverty line (Pew Research Center, 2020). The uncertainty and adversity low-income families are facing put them at higher risk for detrimental short and long-term consequences. It is likely that the COVID-19 crisis will be particularly harmful to very young children of low-income and less-educated parents who are already at higher risk for poorer outcomes. Parents who already experience economic and other stresses may face additional challenges that stack against their ability to provide adequate care and emotional support for their children (Conger et al., 2010; Neppl et al., 2016).

Yet, studies conducted before the COVID-19 pandemic show that low-income families also demonstrate resilience in the face of adversity, which gives us information about the conditions under which risk factors are not associated with negative outcomes (Masten, 2001). In particular, as models of resilience suggest, factors at the individual and family levels such as being positive about the future and feeling supported in the coparenting role may help buffer the negative impact of stress/trauma on families (Masten and Barnes, 2018). Such research in conjunction with work on risk and vulnerability can help guide public policy and intervention efforts to improve the lives of children at risk for maladaptive outcomes (Masten, 2001).

This paper explicitly explores the potential contributions to family resilience during the COVID-19 pandemic. Specifically, we examine the unique importance of pandemic-induced economic hardships (i.e., job and income loss, inability to make ends meet) and pandemic-induced social stressors (i.e., exposure to the virus, loss of childcare) as they relate to parental mental health, parent engagement with children, and children’s socioemotional behaviors among low-income, diverse families. Nascent research on the factors that protect families against the negative effects of the COVID-19 pandemic on family functioning find that parents’ positivity or optimism about the future and feelings of support from their families, including partners, play a key protective role (Li and Xu, 2020; Schug et al., 2021). Therefore, we also examine whether parent positivity and a supportive coparenting relationship protect children and parents from the negative effects of economic and social stressors on family functioning. More specifically, we ask: (1) How are pandemic-induced economic and social stressors uniquely associated with family functioning, including parents’ mental health, parent engagement, and children’s socioemotional behaviors and (2) How do promotive factors such as perceived coparenting support and parents’ positivity moderate the association between pandemic-related stressors and parents’ mental health, parent engagement, and children’s socioemotional behaviors.

### Theoretical Background

We frame this study using a relational developmental systems framework that is commonly used in the field to study resilience (Masten, 2018). Research on resilience has shown that in times of crisis, when individuals experience a high number of risks, people draw on protective processes, including various psychological, social, and economic resources to cope, adapt, or overcome adversity (Masten, 2018). Protective factors are commonly defined as characteristics of the child, family, and broader environment that matter when adversity is high (Masten and Reed, 2002; Masten, 2013; Wright et al., 2013; Masten and Cicchetti, 2016). The interplay between risk and protective factors is central to the concept of resilience, which is defined as the “the capacity of a dynamic system to withstand or recover from significant challenges that threaten its stability, viability, or development” (Masten, 2011, p. 494).

We focus on families with young children because it is a time of unprecedented growth and it is most sensitive to environmental contexts (Shonkoff and Phillips, 2000). As such, economic and social risks or stressors during this period are likely to influence all aspects of development. Although the research is clear that the early years represent an optimal time for brain development, there is less clarity about the factors that might protect children from the adverse effects of poverty and other stressors on family functioning. Research has shown that many children growing up in low-income households are exposed to high quality experiences that promote their development (Cabrera et al., 2007). To bolster these supports, it is critically important to understand the factors during this pandemic that promote children’s social adaptation and family wellbeing in early childhood despite adverse circumstances (Compas et al., 2001). In this study, we examine both the associations between risks and family functioning as well as the promotive factors that might protect children from these negative effects.

A central function of families is to nurture and socialize children to the norms and values of their cultural milieu (Georgas et al., 2001). We focus on the following indicators of family functioning because an extensive body of research has shown them to be significantly and robustly related to young children’s social adjustment: children’s socioemotional skills, parent engagement in learning activities at home, parents’ mental health, coparenting support, and parental positivity (Priel et al., 2019).

The rapid and substantial policy response to the pandemic—extended unemployment insurance and stimulus funds—in many ways protected families from a deeper economic crisis (Ganong et al., 2020). However, little is known yet about the individual- or family-level protective factors that helped people withstand the substantial negative impact of the COVID-19 pandemic on family functioning. Therefore, this study also tests moderation effects.

### Risks Factors and Family Functioning

Risk is typically defined as the elevated probability of a negative outcome that tends to accumulate over time (Evans et al., 2013). The experience of multiple risks is likely to have a cumulative and negative effect on all aspects of family functioning (Evans et al., 2013; Masarik and Conger, 2017). The more risks families experience, the higher the probability of them taking a toll on their wellbeing (Cappa et al., 2011; Crnic and Ross, 2017; de Cock et al., 2017; Rollè et al., 2017). This literature also demonstrates that risks have differential effects on children and families (Griffith et al., 2020; Romero et al., 2020). Families with fewer economic and social resources, as a group, are likely to suffer the most (Duncan and Murnane, 2016). An extensive body of work conducted prior to the current pandemic robustly showed that various types of risk, including economic and social stressors, have short- and long-term effects on all aspects of family functioning, including parents’ mental health, parenting and children’s socioemotional adjustment (McLoyd, 1990; Harvey and Delfabbro, 2004; Fiorini and Keane, 2014; Masarik and Conger, 2017).

The economic and social impact of the COVID-19 pandemic on children and families and the policies implemented to contain the virus, including lock-down orders, school and childcare closures, new regulations for frontline workers—have resulted in multiple sources of risk and stress for families, including but not limited to worries about the future, fear of being infected or becoming terminally ill, pressures related to working under unsafe conditions, and losing childcare arrangements (Pew Research Center, 2020). These economic (e.g., job loss, inability to pay one’s bills) and social sources of stress (e.g., disruption in child care, being exposed to the virus) have the potential to be long-lasting with effects reverberating throughout individuals’ lives. Research to date on the associations between the stress caused by this pandemic and families’ wellbeing has mostly documented economic and health impacts (Brown et al., 2020; Fontanesi et al., 2020; Lawson et al., 2020; Hertz-Palmor et al., 2021). Because the pandemic is still evolving, the science to understand the pandemic’s effects on family functioning, including parenting and mental health, is also unfolding in real time.

Rightly so, early reports of the effects of the pandemic on family functioning have focused on parents of young children (Lawson et al., 2020; Patrick et al., 2020). Decades of research have unequivocally shown that the quality of parenting (e.g., engaging in cognitively stimulating activities, showing love and affection) is critical for children’s development (Smith et al., 2000; Caspi et al., 2004). In times of crisis, parents, especially economically vulnerable parents, may be less responsive and nurturing toward their children which can have dire consequences (Roos et al., 2021). Parents who lost their jobs and childcare arrangements because of the pandemic found themselves spending more time with children at home and having to restructure daily routines and activities to accommodate the new changes (Pew Research Center, 2020). Whether or not the increased parental care time was beneficial for children is uncertain. For some families this increased time at home together may result in more opportunities for learning and structured activities, which support social and cognitive skills development (Cabrera et al., 2020; Gregus et al., 2021). But for other families, the increased time during the COVID-19 pandemic may result in more unstructured and chaotic family organization that increases stress and jeopardizes the quality of parenting (Roos et al., 2021). For many economically vulnerable parents with young children, losing childcare may have meant crowded conditions at home as well as increased demands on parents’ time to cook three meals a day and provide structured activities for their children, which can take a toll on parental mental health. A survey of 405 parents found that about 40% reported major or severe depression and parenting stress during the pandemic (Lee et al., 2021). For parents who were still working during the pandemic, loss of childcare could have presented sizeable challenges in their ability to continue to work. Under these conditions, increased parenting time with children might result in increased stress and diminished positive parenting, with negative implications for children. The same survey conducted by Lee and colleagues found that parents who reported spending an increased amount of time with their children at home also reported higher child anxiety and other behavioral problems (Lee et al., 2021).

A key aspect of family functioning is parents’ mental health (Burke, 2003). The impact of environmental risks on parents’ mental health is well understood. In general, parents who feel they have no control over their lives and are unable to stop worrying are at risk for mental health problems and a more taxing home environment (Conger et al., 2010). Because economic vulnerable parents in the U.S. are already at risk for higher levels of mental health problems (Gard et al., 2020), the added stress caused by the COVID-19 pandemic would likely have a cumulative and negative effect on parents’ mental health. Preliminary research conducted during the COVID-19 pandemic has found increased feelings of stress, depression, and anxiety for parents (Russell et al., 2020; Calvano et al., 2021). The negative effects of pandemic-induced economic stress on parenting and mental health confirms the robust findings on how economic stressors such as job loss and inability to pay one’s bills, more generally, can negatively impact families (Conger et al., 1994).

### Risk Factors and Children’s Socioemotional Development

The development of socioemotional skills (e.g., forming and sustaining relationships with others, experiencing, managing, and expressing emotions) during early childhood is a foundational milestone that supports future learning and development across developmental periods (Sroufe, 2005). Social skills influence children’s self-confidence, empathy, and ability to develop meaningful and lasting friendships and partnerships (Eisenberg and Fabes, 2006). Parents and other caregivers foster socioemotional skills by being affectionate and nurturing and engaging in various activities that provide joy and teach children to take turns, listen, and resolve conflict (Belsky, 1990). Thus, any disruption to the quality of parenting is concerning because it has the likelihood of interfering with this process, with potentially long-term negative consequences for children (Eisenberg and Fabes, 2006). Economically stressed parents have children who exhibit less socially competent children than parents who are better off (Duncan and Murnane, 2016).

However, the association between stress and children’s skill development is not linear. It should be noted that some studies have found that certain types of risk such as parental report of family financial difficulty (on a scale of 0–5) are sometimes associated with increases in Latino/a youth’s prosocial behaviors, especially helping behaviors (Davis et al., 2018, 2020). Helping, sharing, or giving love and support are prosocial behaviors that are intended to benefit others (Padilla-Walker and Carlo, 2014). Studies of young children show that parents teach children to respond with compassion and concern when they witness someone being hurt or expressing a negative emotion such as crying (Farrant et al., 2012; Pastorelli et al., 2015). Studies of Latino families have shown that children are socialized to be caring and nurturing and to exhibit greater concern for others (Eisenberg et al., 2009; Calderón-Tena et al., 2011). Thus, it is possible that stress might be related to increases in children’s prosocial behaviors, especially among Latino families.

In this study, we examine how pandemic-related risk factors such as economic and social stressors are associated with important aspects of family functioning, including parenting behaviors, parents’ mental health, and children’s socioemotional problems and prosocial behaviors.

### Protective Factors and Resilience

Theories of risk and resilience posit that protective factors buffer children from the negative effects of risk and that individuals respond to stress in multiple ways (Putnick et al., 2010; Masten, 2011). Research on stress and resilience has documented how families’ previous adverse experiences provided them with the opportunity to develop effective coping mechanisms that can buffer them from the negative effects of new stressors, such as the current pandemic, on themselves and their children (Schug et al., 2021). Research on what promotive factors are protective in the context of risk in general is not extensive and therefore there is a dearth of information about what types of factors are protective globally and at differing levels of risk (Vanderbilt-Adriance and Shaw, 2008; Masten, 2011). In this study, we test the moderation effects of two factors that have been identified in the emerging COVID-19 literature as being protective: parents’ positivity and feelings of family support, including coparenting support on children’s socioemotional behaviors, parent engagement, and parental mental health (Li and Xu, 2020; Schug et al., 2021).

Pre-pandemic research shows that individuals who are high in positivity have better physical health, higher levels of emotional well-being, more positive social relationships, and improved capacity to cope with a broad range of stressful situations (Brissette et al., 2002; Assad et al., 2007; Kochanska et al., 2007; Carver et al., 2008; Baumgartner et al., 2018). Research with low-income ethnic minority mothers has shown that maternal positivity is associated with lower levels of maternal internalizing symptoms and higher levels of child adjustment (Taylor et al., 2010, 2012). In both mothers and fathers, positivity has been related to effective parenting and children’s socioemotional adjustment (Jeon and Neppl, 2019). There is also some evidence that parental positivity acts as a buffer against the negative impact of economic stress on parents’ mental health (Taylor et al., 2010, 2012). A study conducted in Germany during the pandemic found that in a large sample of healthcare workers, optimism was significantly associated with lower scores of depressive and anxiety symptoms (Schug et al., 2021). Overall, the literature suggests that positivity helps maintain positive parenting during adverse times and may serve as a psychological resource against the negative effects of economic stress on parents and children. However, the roles of positivity and other family supports during this pandemic have yet to be explored. We thus examine whether coparenting support and parent positivity are not just promotive of good outcomes but also protective, facilitating better parenting interactions with children and better child adjustment.

Coparenting or the ability of couples to work together as a team to manage their parenting responsibilities is a significant promotive aspect of family functioning (Feinberg, 2003; McHale, 2007). The quality of the coparenting relationship has been shown to be one of the strongest factors associated with children’s social adjustment (e.g., Cabrera et al., 2012; Palkovitz et al., 2013; Gallegos et al., 2017; Choi and Becher, 2018; Mack and Gee, 2018) and with mothers’ and fathers’ positive parenting behaviors (Cabrera et al., 2009, 2011; Morrill et al., 2010). In one study in New Zealand conducted during the pandemic, researchers found that the association between depression and negative quality of parenting was found only for couples who reported low levels of coparenting support (McRae et al., 2021). Similarly, a study of 1,547 Chinese parents (age range=12–60years) showed that family support (assessed as using the family support subscale of the Multidimensional Scale of Perceived Social Support) was protective in maintaining mental health (Li and Xu, 2020). It may be that being in a supportive coparenting relationship mitigates the demands that parenting during a pandemic may place on parents. Moreover, parents who feel supported by their co-parent may feel greater confidence in their ability to parent, particularly during a stressful period of time such as the COVID-19 pandemic.

### Current Study

Based on this extant literature, we have two research questions. First, how are pandemic-induced economic and social stressors uniquely associated with indicators of family functioning such as parents’ mental health, parent engagement, and children’s socioemotional problems and prosocial behaviors? Second, how do promotive factors, such as perceived coparenting support and parent positivity, moderate the associations between pandemic-related stressors and indicators of family functioning? Based on models of risk and resilience, we hypothesize that parents who report a high number of economic and social stressors will also report more depressive symptoms and stress, less parent engagement than pre-COVID period (main effects). Because the association between economic stress and child socioemotional behaviors is inconsistent in the literature, we do not specify a direction for this hypothesis. We also hypothesize that parents high on positivity and enjoying high levels of coparenting support will report fewer depressive symptoms and stress, more frequent engagement, and more prosocial behaviors in their children than parents with low levels of positivity and coparenting support (main effects). Given the state of the empirical evidence, we do not hypothesize about the relative importance of each set of stressors. Finally, we hypothesize that the association between economic and social stressors and family functioning outcomes will be reduced when parents have high levels of supportive coparenting relationship and high levels of positivity (interaction effects).

## Materials and Methods

### Procedures

Data were collected from a sample of first-time parents participating in a NICHD-funded longitudinal intervention study (BabyBooks 2 project, BB2) that aimed to give information about child development to low-income parents (removed for blind review). Participating families were recruited from centers that administer the Specific Supplement Nutrition Program for Women, Infants, and Children, health care clinics, emergency department waiting rooms, parks, and community centers in both the Washington, DC metropolitan area and in Orange County, California. To be eligible for the BB2 intervention project, parents had to be first-time parents of a baby less than 9months of age; be cohabiting, over the age of 18; making less than $75,000 per year; and, literate at a first-grade reading level in either English or Spanish. All infants were full term (37weeks of gestation or longer). Families were told that the project was aimed at understanding how reading to babies helps them learn and were offered children’s books and compensation for their time.

From May to August 2020, eligible parents in the BB2 project were contacted *via* text message about their interest in participating in an online survey study about their COVID-19-related experiences. The survey was hosted on Qualtrics^1^ (Qualtrics, Provo, UT), an online survey tool that allows to create, distribute and record survey questions. Once parents consented to participate, they received a personal link to access the survey on their phone; only one parent requested to take the survey on a computer. Parents received either an English or Spanish version, based on their preferred language, and were given a 21-day timeframe to complete the survey. Of the total BB2 sample, 292 parents were still actively enrolled at the time of this survey. All 292 parents were contacted and 247 consented to participate (84.6% of response rate). All data were collected between July 2020 and September 2020. Each participant was compensated with a $20 e-gift-card or cash and was entered in a raffle to win one of four $50 e-gift-cards. To reduce missing data, participants were reminded to complete each survey question automatically by the online survey software. After viewing the reminder, participants were allowed to skip items if they chose to do so. No identifying information was collected during the survey. The personalized survey link was used to match demographic information from the database. Participants spent an average of 26min to fully complete the survey. Our final sample consisted of 247 parents from 142 families, of which 210 parents were a couple. The remaining 37 parents were 32 mothers and 5 fathers whose partners either did not want to participate or could not.

### Participants

All of the participants were low-income parents with their children between the ages of 22 and 55months (Mean age=2.9years, SD=0.5) at the time the COVID-19 survey was administered; 48.6% of the total sample (*n*=120 parents) resided in the greater Washington, D. C. metropolitan area including Virginia and Maryland and 51.4% resided in Orange County, California. Forty-four percent (*n*=108) of the children were boys and 56% (*n*=136 children) were girls. The sample included slightly more mothers (55.5%; Mean age=30.0years old, SD=5.8) than fathers (44.5%; Mean age=32.7years old, SD=6.7). The analytic sample (*n*=142) did not significantly differ from the full BB2 sample (*n*=240) on household income or education levels assessed as the highest education level in the family. The average annual household income before the pandemic started was USD $40,051 (SD=25,172). Though the bilingual (Spanish-English) BB2 study’s participants are predominately Hispanic, our response rate was greater for Hispanic parents (70%). Table 1 demonstrates the sample demographics and descriptive data of study variables by parent gender.

**Table 1:** Sample Demographics and Descriptive Data by Families and Parent Gender.

	Combined (*n*=247)		Fathers (*n*=110)		Mothers (*n*=137)	
*Demographics*	M(SD)/%	*n*	M(SD)/%	*n*	M(SD)/%	*n*
Parents’ Education						
Less than high school	11.7%	29	21.8%	24	3.6%	5
High school diploma	19.4%	48	18.2%	20	20.4%	28
Some college	30.8%	76	29.1%	32	32.1%	44
2–4year college	12.1%	30	11.8%	13	12.4%	17
4year college or above	25.9%	64	19.1%	21	31.4%	43
Parents’ Ethnicity						
White	7.3%	18	9.1%	10	5.8%	8
Black	13.8%	34	12.7%	14	14.6%	20
Hispanic	70.4%	174	66.4%	73	73.4%	101
Others	8.5%	21	11.8%	13	5.8%	8
Parent age (in years)	31.2(6.3)	245	32.7(6.7)	109	30.4 (5.8)	137
	Families (*n*=142)		Fathers (*n*=110)		Mothers (*n*=137)	
*Study Variables*	M(SD)	Range	M(SD)	Range	M(SD)	Range
Parent Mental Health	–	–	5.2(3.7)	0–19	6.5(3.6)	0–18
Parent Engagement	39.1(6.0)	10–50	18.4(3.8)	5–25	20.5(2.9)	8–25
Child Socioemotional Problems	2.5(1.0)	0–4.8	2.4(1.2)	0–4.8	2.5(1.3)	0–5
Child Prosocial Behaviors	4.0(0.8)	0–5	4.0(0.9)	0–5	4.1(0.8)	2–5
Economic Stressors	1.0(0.7)	0–2	0.9(0.8)	0–2	1.0(0.8)	0–2
Social Stressors	0.6(0.6)	0–2	0.6(0.6)	0–2	0.6(0.6)	0–2
Parent Positivity	22.4(3.9)	10–30	22.7(4.0)	10–30	22.2(4.9)	9–30
Coparenting Support	34.2(7.5)	2–42	35.6(7.2)	9–42	34.1(8.4)	2–42

*Due to missing data on some variables, not all responses to individual items sum to 247 participants or 142 families* (Larose et al., 2021).

### Measures

The predictor variables include four stressful experiences related to the pandemic in both economic and social domains. The economic factor consistsw of self-reported ratings of changes in employment and financial ability to make ends meet since the COVID crisis began. The social factor consists of self-reported ratings of exposure to the SARS-CoV-2 virus and difficulty in accessing childcare since the COVID crisis began. The outcome variables include four key aspects of family functioning: parental mental health, parent engagement, and parent report of changes in child’s socioemotional problem behaviors and prosocial behaviors during the pandemic. We also examined two protective factors (moderators) that are likely to buffer the stressful experiences brought by the pandemic on family functioning. The predictors, outcome variables, and moderators are described in detail below as well as in Table 1.

#### Economic and Social Stressors

We asked participants about changes in four stressful experiences closely related to economic and social life experienced since the national outbreak of SARS-CoV-2 (adapted from Brailovskaia and Margraf, 2020). The survey included four items: (1) job or income loss (2) inability to make ends meet (3) exposure to SARS-CoV-2 virus (4) difficulty accessing childcare. Table 2 shows the number and percent of families who reported negative impact in these aspects. Responses to questions about income loss and inability to making ends meet were summed into an economic stressor variable. Similarly, we summed both virus exposure and difficulty accessing childcare into a social stressor variable. Both economic and social stressors were entered as ordinal variables (0=no negative impact, 1=negative impact in one aspect, 2=negative impacts in two aspects) for each parent.

**Table 2:** Number and Percent of Parents Encountering the COVID19-related Stressors.

Types of Economic stressors	*N* (total=242)	%	Types of Social stressors	*N* (total=244)	%
No stress	86	35.5%	No stress	116	47.5%
Job loss only	28	11.6%	Expose to virus only	40	16.4%
Inability to make ends only	59	24.4%	Daycare disruption only	67	27.5%
Both job loss and inability to make ends meet	69	28.5%	Both exposure to virus and daycare disruption	21	8.6%

*Due to missing data on some variables, not all responses to individual items sum to 247*.

##### Job or Income Loss

Participants were asked about changes in their employment status since the pandemic began and could choose from “No change,” “Lost job/Lost hours” or “Got new job/Gained hours” (Larose et al., 2021). Lost job/h was coded as 1 and no change and new job/gained hours as 0.

##### Inability to Make Ends Meet

Participants were asked about changes in their “ability to pay bills” and “ability to buy basic needs” and could choose from “No change,” “Yes, it is easier than before,” “Yes, it is slightly more difficult,” and “Yes, it is much more difficult.” For each item, when participants reported some level of difficulty, they were scored as 1, otherwise they were scored as 0.

We summed job loss/work hours loss and financial struggles to create an economic stressors variable at the individual level ranging from 0=no economic stress, 1=one economic stressor, to 2=two economic stressors.

##### Exposure to SARS-CoV-2 Virus

Participants reported to what extent they or the people around them (e.g., family members, close co-workers) had been affected by the COVID-19 pandemic (e.g., “I have tested positive myself” or “Someone with whom I live or work tested positive”). The answers from both questions were merged in a single variable, named “Exposure to virus” and transformed into a dichotomous variable (0=No, 1=Yes). Participants were considered not exposed (exposure=0) if they did not endorse the exposure to virus items and instead reported “My physical health has not been affected” and also “The health of those close to me has not been affected.” If one of these was not selected (e.g., not positive themselves but people close to them were infected), they were considered as being exposed to the SARS-CoV-2 virus (exposure=1).

##### Disruption to Childcare

Mothers and fathers were asked changes in their access to childcare since the pandemic began and could select from “No change,” “Yes, it is easier than before,” “Yes, it is slightly more difficult,” and “Yes, it is much more difficult.” If “No change” or “easier than before” was selected, they were scored as 0; otherwise, they were scored as 1.

We summed the virus exposure and difficulty accessing childcare to create a social stressors variable at the individual level ranging from 0 to 2.

We then created parent-level economic and social stressors scores. When both parents in the same family responded, an average score was used. When only one parent in the family responded, that parent’s score was used to represent both parents. Therefore, the parent-level economic and social stressors also ranged from 0 to 2 with 5 possible levels. For example, if a parent-level economic stressors score=0, it means neither parent reported negative change in employment status or financial ability since the COVID; 0.5=one parent reported negative change in employment status or financial ability; 1=one parent reported negative changes in employment status and financial ability or both parents reported one negative change, 1.5=one parent reported a negative change and the other parent reported two negative changes, 2=both parents reported negative changes in employment status and financial ability. These scores were entered as continuous variables in later analyses.

#### Parents’ Mental Health

Parental mental health during the pandemic was assessed with three items about perceived anxiety and depression, adapted from the Patient Health Questionnaire, PHQ-4 (Kroenke et al., 2009) and four items of perceived life stress, adapted from the Perceived Stress Scale, (PSS; Cohen et al., 1983).

##### Depression

Each parent rated how often they have been bothered over the last two weeks on a 4-pt Likert scale with 0=“Never,” 1=“Sometimes,” 2=“Fairly often,” and 3=“Very often.” Items include “Not being able to stop or control worrying.,” “Feeling down, depressed, or hopeless.,” and “Little interest or pleasure in doing things.” One item “Feeling nervous, anxious or on edge” from the PHQ-4 scale was not included in this survey because there was no variability everyone responded feeling anxious.

##### Stress

Each parent rated how often they experienced stressful situations in the past month. We used 4 items from the PSS to measure the degree to which situations in one’s life are appraised as stressful (Cohen et al., 1983). The shortened scale was highly correlated with the original 14-item scale. Participants were assigned 0=“Never,” 1=“Sometimes,” 2=“Fairly often,” and 3=“Very often” for each of the questions included. These questions asked how often in the past month (1) “you were unable to control the important things in your life”; (2) “things were going your way” (reverse coded); (3) “confident about your ability to handle your personal problems?” (reverse coded) and (4) “difficulties were piling up so high….”

These seven items were added up to a mental health score that ranged from 0 to 21. Higher scores indicate more depressive symptoms and feeling more stressed. The Cronbach’s alpha was 0.75, which indicates an acceptable level of internal consistency for the combined scales with the study sample. Mothers’ reports of mental health scores and fathers’ reports of mental health scores were treated separately because they were not significantly different from each other (*r*=−0.01) and because this is a meaningful characteristic of individual’s functioning.

#### Parent Engagement

Parents were asked about how often they were doing some specific activities with their child since the COVID-19 pandemic began using a 6-point scale (1=“not at all,” 2=“Rarely,” 3=“a few times a month,” 4=“a few times a week,” 5=“about once a day,” 6=“more than once a day”). Items include: Playing together, putting the child to bed, going for a walk together, singing songs and telling stories, and reading a book together. The Cronbach’s alpha was 0.72, indicating an acceptable level of internal consistency for this measure. Summary ratings of 5 items range from 5 to 30. Higher scores indicate more engagement in these reported activities. We used the sum score of mothers’ reports of parent engagement and fathers’ reports of parent engagement to assess both parents’ total engagement time spent with the child at home. This is an improvement over past studies that assess total parenting behaviors with only one parent, typically mothers. In addition, mothers ‘and fathers’ reports of engagement were correlated (Pearson r=0.19). Thus, we used sum score to capture the total amount of children’s “exposure” to parenting from their mothers and fathers. When only one parent in the family responded, we took that parent’s score to represent total parenting.

#### Child Socioemotional Behaviors

We modified questions from the problems and competence subscales from the Brief Infant and Toddler Socioemotional Assessment (BITSEA; Briggs-Gowan and Carter, 2002) and developed new answer choices to capture parents’ perceptions of changes in children’s behaviors since the COVID-19 pandemic began.

##### Child Socioemotional Problems

Mothers and fathers were asked to rate on a 5-point Likert scale (1=“a lot less,” 2=“a little less,” 3=“the same,” 4=“a little more,” 5=“a lot more,” and “does not apply”) how much their child’s behavior has changed as compared to before the COVID began. Five types of behaviors were assessed: (1) “been having tantrums and angry outbursts”; (2) “been struggling to manage their emotions”; (3) “been engaging in aggressive behavior such as hitting, biting, scratching and throwing objects…”; (4) “been crying”; and (5) “been needing to be held.” “Does not apply” was coded as 0. Ratings of these items were averaged and the scores range from 0 to 5. The Cronbach’s alpha was 0.86, indicating a good level of internal consistency for this measure.

##### Child Prosocial Behaviors

Prosocial behaviors included three items rated on a 5-point scale as above (1=“a lot less” to 5=“a lot more”) and “Does not apply.” These included: As compared to before the COVID began, has your child (1) “been talking/communicating with you”; (2) “been wanting to help”; (3) “been affectionate (e.g., gives hugs, uses caring words, etc.).” “Does not apply” was coded as 0. Ratings of these items were averaged and the scores range from 0 to 5. Internal consistency was adequate with Cronbach’s alpha of 0.65.

When both parents in the same family responded, an average score was used. When only one parent in the family responded, that parent’s score was used to represent both parents.

#### Parent Positivity

To assesses positivity during the pandemic, we included 6 items from the Positivity Scale (P Scale) that includes self-esteem, life satisfaction, and positivity (Caprara et al., 2012). Sample items include “I have great faith in the future” and “I look forward to the future with hope and positivity.” Participants rated their agreement on a 5-point Likert scale (1=“strongly disagree,” 2=“disagree,” 3=“neither,” 4=“agree,” 5=“strongly agree”). Item 6 (“At times, the future seems unclear to me”) was reverse coded before running the analyses. The total score ranges from 6 to 30. The higher scores indicate being more positive or hopeful about the future. Responses had good internal consistency (Cronbach’s alpha=0.79). Because mothers’ and fathers’ reports of positivity scores were correlated (Pearson *r*=0.25) and did not differ, mothers’ and fathers’ scores in the same family were averaged to create parent scores. Reports from single-respondent families were used as parent scores.

#### Coparenting Support

To assess perceptions of coparenting support during the pandemic, we used the seven items on the Coparenting Support subscale from the brief Coparenting Relationship Scale (CRS; Feinberg et al., 2012). Items such as “my partner appreciates how hard I work at being a good parent” were rated on a 7-point scale (0=“not true of us” to 6=“very true of us”). Summary scores range from 0 to 42. The higher scores indicate more support from the other parent. Responses were averaged and had good internal consistency (Cronbach’s alpha=0.89). Because mothers’ and fathers’ reports of coparenting scores were correlated (Pearson r=0.24) and did not differ, mothers’ and fathers’ scores in the same family were averaged to create parent scores. Reports from single-respondent families were used as parent scores.

### Analytic Plan

The analytic sample consisted of 142 families, including 137 mothers and 110 fathers. For our study variables, less that 2% of data were missing at the parent, including one missing score for parent positivity, and two for coparenting support.

We conducted one path analysis with maximum likelihood (ML) method to calculate estimators using RStudio 1.2.5^2^ (PBC, Boston, MA). In the model we allowed the predictors and the outcomes to covary. The model included 4 parent-level predictors (economic stressors, social stressors, parent positivity, and coparenting support), 5 outcomes (maternal mental health difficulties, paternal mental health difficulties, total parent engagement, child socioemotional problems, and child prosocial behaviors), and 1 control variable (highest education level in the family). To examine interaction effects, we added 4 interactions (economic stressors × parent positivity, economic stressors × coparenting support, social stressors × parent positivity, social stressors × coparenting support) in the model. Both main effect and moderation effect models were saturated. The four main predictor variables were first mean-centered and then used to calculate the interactions to reduce multicollinearity among the predictors. We reported standardized estimates of all estimators. Finally, we used simple slopes analysis to visualize the moderation interactions using Process v3.5 in SPSS 27 (Hayes, 2012).

## Results

### Descriptive and Correlation Analyses

Among the 142 families in our sample, 77.5% percent of families reported negative change in levels of economic hardship and 63% reported experiencing at least one social stressor since the pandemic. Parent report of each set of stressors is presented in Table 2. Parents reported high level of positivity during the pandemic (Mean=22.4, SD=3.9) and high level of supportive coparenting relationship since the pandemic (Mean=34.2, SD=7.5). Mothers (Mean=6.5, SD=3.6) and fathers (Mean=5.2, SD=3.7) reported low levels of depression, anxiety and stress during the pandemic. Mothers and fathers reported more engagement with their child since the pandemic began (Mean=39.1, SD=6.0). Families reported no change in their children’s socioemotional problems since the pandemic began (Mean=2.5, SD=1.0); and, reported observing more prosocial behaviors in their children since the pandemic began (Mean=4.0, SD=0.8). Mean, standard deviation, range are presented in Table 1 and correlations of study variables are presented in Table 3.

**Table 3:** Zero-order Correlations for All study Variables Aggregated except for Parental Mental Health.

	Study Variables	1	2	3	4	5	6	7	8	9
1	Economic stressors	–								
2	Social stressors	0.19^*^	–							
3	Parent positivity	−0.10	−0.07	–						
4	Coparenting support	0.01	−0.14	0.42^**^	–					
5	Mothers’ mental health	0.15	0.08	−0.46^**^	−0.26^**^	–				
6	Fathers’ mental health	0.25^**^	0.06	−0.35^**^	−0.18	−0.01	–			
7	Parent engagement	−0.17	−0.34^**^	0.17^*^	0.31^**^	−0.01	−0.10	–		
8	Child socioemotional problems	−0.03	0.19^*^	−0.07	−0.03	0.32^**^	−0.03	0.05	–	
9	Child prosocial behaviors	0.21^**^	0.06	0.24^**^	0.26^**^	−0.11	−0.01	0.03	0.17^*^	–

*Due to missing data on some variables, not all responses sum to 142.*

* *p<0.05;*

** *p<0.01*

### Path Analysis: Main Effects

**Figure 1:**
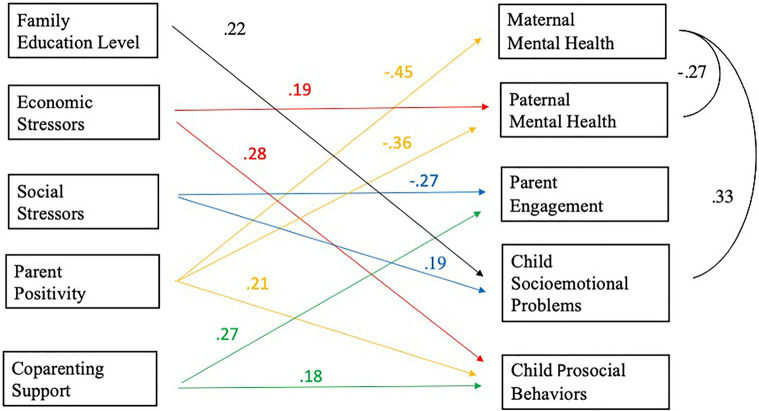
The roles of Economic stressors, Social stressors, Parent positivity, and Coparenting support on maternal and paternal mental health, Parent engagement, Child socioemotional problems and Child prosocial behavior controlling for family education level. *Note*. All predictors are mean-centered. For parsimony, errors and non-significant coefficients are omitted from the figure. All standardized coefficients and covariances are significant at p < 0.05. Significant paths are color-labeled based on the predictors.

We conducted one path model to examine the associations between parent risk factors (i.e., economic and social stressors) and the five outcomes (i.e., mother and father mental health, parent engagement, child socioemotional problems and prosocial behaviors; Figure 1).

Parents’ increase of economic stressors since the pandemic began was significantly associated with parent report of more children’s prosocial behaviors, controlling for family education level. That is, one standard deviation increase in economic stressors was associated with a 0.28 standard deviation increase in child prosocial scores (beta=0.28, 95% CI=[0.*12, 0.44*], *p*<0.01), keeping everything else constant. Parents’ increase of economic stressors since the pandemic began was positively and significantly associated with paternal (but not maternal) mental health scores during the pandemic (beta=0.19, 95% CI=[0*.02, 0.37*], *p*<0.05), controlling for family education level. That is, one standard deviation increase in economic stressors was associated with 0.19 standard deviation increase in paternal mental health scores, keeping everything else constant.

Parents’ increase in social stressors since the pandemic began was significantly associated with less parent engagement (beta=−0.27, 95% CI=[*−0.42, −0.13*], *p*<0.001) and with more parent-reported socioemotional problems in the child as compared to before the pandemic (beta=0.19, 95% CI=[0.*03, 0.33*], *p*<0.05), controlling for family education level. That is, one standard deviation increase in social stressors was associated with 0.27 standard deviation decrease in parent engagement score and 0.19 standard deviation increase in child socioemotional problem scores, keeping everything else constant.

During the pandemic, parent positivity showed negative association with mothers’ mental health difficulties scores, beta=−0.45, 95% CI=[*−0.60, −0.30*], *p*<0.001, and fathers’ mental health difficulties scores, beta=−0.36, 95% CI=[*−0.54, −0.19*], *p*<0.001), as well as positive association with parent-reported children’s prosocial behaviors since the pandemic began, beta=0.21, 95% CI=[0.*05, 0.38*], *p*<0.05, controlling for family education level. That is, one standard deviation increase in parental positivity score was associated with 0.45 standard deviation decrease in maternal mental health score and 0.36 standard deviation decrease in paternal mental health score, and associated with 0.21 standard deviation increase in children’s prosocial behaviors score, keeping everything else constant.

Since the pandemic began, more coparenting support was associated with increased parent engagement, beta=0.27, 95% CI=[0*.11, 0.43*], *p*<0.01, and parent report of increased prosocial behaviors (beta=0.18, 95% CI=[0.*02, 0.35*]*, p*<0.05), controlling for family education level. That is, one standard deviation increase in coparenting support score was associated with 0.27 standard deviation increase in parent engagement score and 0.18 standard deviation increase in the prosocial behaviors score, keeping everything else constant.

### Moderation Effects

To test the moderation effects, four interaction terms (economic stressors × parent positivity, economic × coparenting support, social stressors × parent positivity, social stressors × coparenting support) were added to the main effects model. We report three significant interactions.

**Figure 2:**
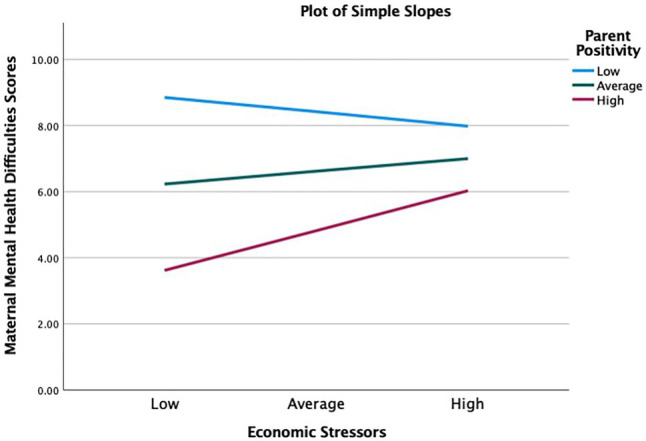
Parent positivity moderating the effect of economic stressors on maternal mental health. High=1 standard deviation above the sample mean, average=sample mean, and low=1 standard deviation below the sample mean.

First, parent positivity during the pandemic, a protective factor, moderated the association between increases in economic stressors since the pandemic and maternal mental health scores during the pandemic, beta=0.26, 95% CI=[0*.09, 0.42*], *p*<0.01. The positive association between economic stressors and maternal mental health difficulties was reduced for mothers who lived in homes with high levels of parent positivity. Simple slopes analysis (Figure 2) showed that, mothers’ mental health difficulties were the highest at low level of parent positivity (−1 SD). At the low level of parent positivity, the association between economic stressors and maternal mental health was negative and non-significant (*b*=−0.63, s.e.=0.57, *p*=0.27). At the average level (sample mean), the relationship was positive and non-significant (*b*=0.57, s.e.=0.40, *p* =0.16). Finally, at high level of parent positivity (+1 SD), the relationship was positive and significant (*b*=1.76, s.e.=0.57, *p*<0.01). So, for low and average levels of positivity, mothers’ mental health difficulties remained higher independent of the number of economic stressors. Meanwhile, high level of parent positivity kept mothers’ mental health difficulties lower, even though this protective effect became weaker as economic stressors accumulated.

**Figure 3:**
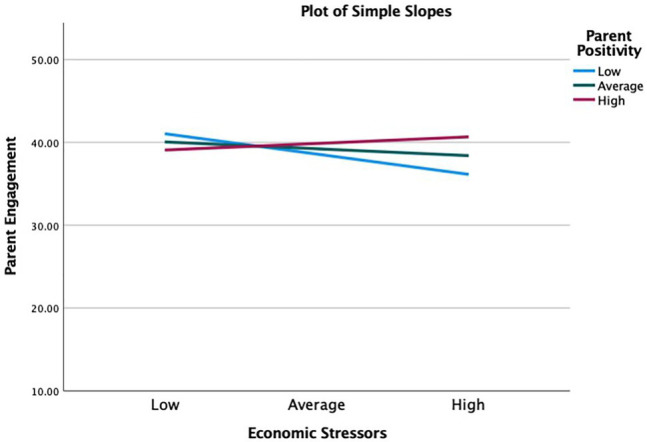
Parent positivity moderating the effect of economic stressors on maternal mental health. High = 1 standard deviation above the sample mean, average = sample mean, and low = 1 standard deviation below the sample mean.

Second, parent positivity during the pandemic, a protective factor, moderated the association between increases in economic stressors since the pandemic and parent engagement during the pandemic, beta=0.27, 95% CI=[0*.11, 0.44*], *p*<0.01. Simple slopes analysis (Figure 3) showed that, parent engagement were the highest at low level of parent positivity (−1 SD). At the low level of parent positivity, the association between economic stressors and parent engagement was negative and significant (*b*=−3.6, s.e.=0.99, *p*<0. 001). At the average level (sample mean), the relationship was negative and non-significant (*b*=−1.2, s.e.=0.71, *p*=0.09). Finally, at high level of parent positivity (+1 SD), the relationship was positive and non-significant (*b*=1.15, s.e.=1.03, *p*=0.27). So, for low and average levels of positivity, parent engagement decreased as the number of economic stressors accumulated. For high level of positivity, parent engagement increased as economic stressors accumulated. Therefore, average or high level of parent positivity buffered parents from the negative effect that economic stressors had on parent engagement.

**Figure 4:**
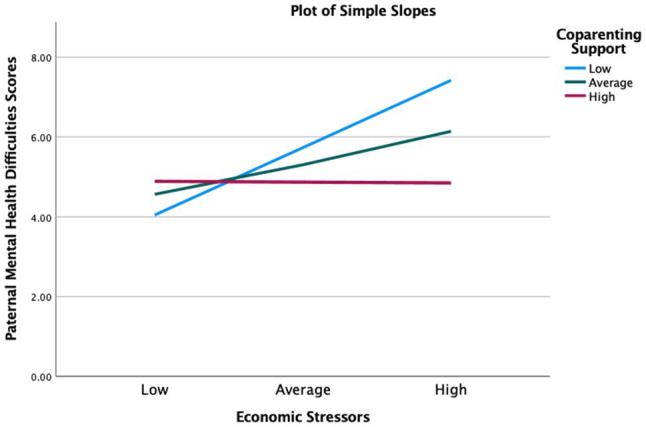
Coparenting support moderating the effect of economic stressors on paternal mental health. High=1 standard deviation above the sample mean, average=sample mean, and low=1 standard deviation below the sample mean.

Third, more coparenting support since the pandemic began, a protective factor, moderated the association between increases in economic stressors since the pandemic began and more paternal mental health difficulties during the pandemic, beta=−0.30, 95% CI=[*−0.54, −0.06*], *p*<0.05. The association between economic stressors and fathers’ mental health difficulties was significantly reduced for fathers who lived in homes with high levels of coparenting support. Simple slopes analysis (Figure 4) showed that, at low level of coparenting support (−1 SD) during the pandemic, the association between economic stressors and paternal mental health was positive and significant (*b*=2.50, s.e.=0.61, *p*<0.001). At the average level (sample mean), the association was also positive and significant (*b*=1.23, s.e.=0.49, *p*<0.05). Finally, at high level of coparenting support (+1 SD), the association was negative and non-significant (*b*=−0.03, s.e.=0.71, *p*=0.97). So, for low and average level of coparenting support, fathers’ mental health difficulties increased significantly as family economic stressors accumulated. However, at the high level of coparenting support, fathers’ mental health did not increase as economic stressors accumulated. Therefore, high level of coparenting support buffered fathers from the negative effect that economic stressors had on fathers’ mental health difficulties.

## Discussion

The ongoing pandemic has waned in some parts of the world but continues to devastate many communities world-wide. In its wake, it has left a trail of destruction and suffering with as of yet unknown long-term consequences. The results from our analysis can help us understand the impact of pandemic-related economic and social stressors on family functioning in an economically and diverse sample of families. We examine both risks and protective factors, which can help policymakers and practitioners allocate resources judiciously and build on the resilience of these families to support their wellbeing. First, our data show that about 40% of low-income parents reported job loss and more than half of these parents struggle to their make ends meet due to the pandemic. Approximately a quarter of our participants have had some exposure to the virus and more than a third had no access to childcare (see Table 2). Given that the sample was predominately Latino and data were collected in the summer of 2020, these rates of COVID-19 exposure were likely modest in comparison to rates now in which Latinos are a disproportionately larger number of cases and fatalities in the U.S (Center for Disease Control and Prevention, 2021). Overall, our results align with other surveys of parents during the pandemic that have found that the majority of mothers and fathers surveyed reported increased financial strain and more than a third experienced increased social stress (Brown et al., 2020; Gassman-Pines et al., 2020). As studies conducted with international samples have shown, the economic stresses of COVID-19 crisis have worsened parents’ mental health and stress, especially for fathers in our sample, but also increased children’s prosocial responses to the economic stress experienced by their parents (Francisco et al., 2020; Golding et al., 2021; Westrupp et al., 2021). But, as we show in this study, there are subtle but important differences in the ways mothers, fathers, and children’s wellbeing have been affected by the pandemic.

There are four key findings of the present research. First, consistent with our hypothesis, we find that half a year into the pandemic parents experienced similar increases in risk factors – social and economic stressors--with similar consequences for family functioning, but with some exceptions. Only fathers reported more mental health difficulties during the pandemic in response to increased economic risk. But, both parents who reported more economic stressors since the pandemic also observed that their children behaved more prosocially (e.g., wanting to help). These findings seem counterintuitive but they are consistent with studies showing that children are taught at a young age to respond with concern and love when they see someone in distress. In this sense, these findings align with previous work with Latinx families with adolescent children, in which parental financial strain was associated with increases in youth’s prosocial behaviors, especially helping behaviors (Davis et al., 2018, 2020). In a more recent study of parents of children of 8years and younger, COVID-19 pandemic-related financial and mental health stresses were similarly associated with increases in children’s prosocial behaviors (removed for blind review). The contribution that children’s positive reactions can have on family functioning needs to be considered, supported, and encouraged.

Second, both parents reported experiencing similar increases in social stressors (i.e., exposure to COVID and disruption in childcare arrangements) since the pandemic began with similar negative repercussions for family functioning. Unexpectedly, we found no adverse effects specifically on mothers’ or fathers’ mental health among the parents in our sample who reported pandemic-induced social stressors. Being exposed to the virus or having no access to childcare for their young children did not significantly worsen their mental health or the perception they had of their children’s prosocial behaviors. However, as expected, it substantially undermined their perception that their children were misbehaving such as having more tantrums, and engaging in aggressive behaviors such as hitting. And social stressors were related to less time spent with their children in fun activities such as playing or reading. Disruption of childcare arrangements and more COVID-19 contact likely depleted parents’ reserves and increased stress, which can influence children’s behaviors and parents’ risk for negative parenting such as maltreatment (Brown et al., 2020). In a study of the protective benefits of childcare, Larose and colleagues (2020) found that for families experiencing adversity, parent care was associated with more child externalizing behaviors as compared to childcare attendance.

Although parents who are stressed tend to perceive their children’s behavior more negatively, it is also the case that children who are in very stressful situations might have a difficult time coping with sudden changes. The pandemic completely and abruptly changed the childcare environments for many children. It is likely that children who could not understand why they are not able to attend childcare and interact with their friends and teachers as they did before the pandemic, might throw more temper tantrums and be irritable to show their frustration. Children’s misbehaviors might also indicate more pandemic-induce family conflict or because everyone is at home at the same time parents have more engagement with their children and more opportunities to witness more problematic behaviors. The connections among social stressors, lower parental engagement, and more problematic behaviors in children are worrisome because the pandemic is ongoing and many parents are dealing numerous contextual challenges such as the aftermath of a COVID-19-related sickness or death due or continued inability to find affordable and consistent childcare.

Our findings are generally consistent with past studies that parents who experience a lot of stress tend to behave less positive toward their children (Brown et al., 2020; Calvano et al., 2021) and extend this literature by showing that some social stressors, in particular changes in childcare arrangements, have negative consequences for fathers, and not just mothers. Feeling anxious and stressed out about getting the virus, passing it to their families, and not having a safe and reliable place for their children have potentially detrimental effect on mothers and fathers with dire consequences for children. Disruption of childcare arrangements and more COVID-19 contact likely depleted parents’ reserves and increased stress, which can influence children’s behaviors and parents’ risk for negative parenting such as maltreatment (Brown et al., 2020). In a study of the protective benefits of childcare, Larose and colleagues (2020) found that for families experiencing adversity, parent care was associated with more child externalizing behaviors as compared to childcare attendance. Programs and policies need to prioritize supporting fathers and mothers by ensuring that reliable and high-quality care and acknowledging that the childcare is also a “father issue.”

Third, the silver lining in these findings is that in addition to the real increases in risks experienced by our families, they also reported strengths – or promotive factors – that could help them get through these difficult times. In general, both parents reported similar strengths with one exception. As hypothesized, parents who reported feeling more positive about the future also reported that they felt less stressed and depressed during the pandemic and observed that their children had more prosocial behaviors compared to before the pandemic. Maintaining a positive attitude and hope for the future has shown to be associated with less depression, more adaptation, and general good outcomes in adults (Taylor et al., 2010, 2012; Schug et al., 2021). Programs should build into their services specific attention not just to decreasing depressive symptoms but also to supporting and maintaining positivity and hope for the future.

Another source of support and strength for our families was the support they gave each other in their role as parents. Consistent with our hypothesis, parents who reported a more supportive coparenting relationship compared to before the pandemic also observed more prosocial behaviors in their children and reported engaging in more activities such as reading, or playing with their children since the pandemic began. An extensive literature has consistently shown that parents who support each other as parents are more likely to have better outcomes for themselves and their children (Cabrera et al., 2009; Palkovitz et al., 2013; Choi and Becher, 2018; McRae et al., 2021). Our findings contribute to this literature and show interdependence of family functioning: improving parent–parent relationship spills over in beneficial ways to the father- and mother–child relationships. Collectively, these results suggest that early on in the pandemic, families were trying to cope with these social and economic stressors and that families without economic help would likely continued to feel less positive and perhaps decreased their engagement with children. Given that possibility and our results, policymakers and programs need to support parents’ mental health as well as provide economic relief (McFarlane et al., 2017).

Finally, as hypothesized, we identify two dimensions of family dynamics that seem to *protect* families against the adverse effects of COVID-19-related stressors on their wellbeing. Identifying stressors and how they impact family functioning is critically important, but it is just as critical to identify the support systems that families have in place to help them deal with adverse situations. Consistent with our hypotheses, we find evidence that parent positivity and coparenting support, promotive factors -- are instrumental in helping parents stay less stressed and anxious. In other words, these factors protect parents from the negative effects of stress on their mental health. And, here again, we find different protective factors for fathers and mothers. We find strong evidence that the negative association between economic stressors and fathers’ mental health difficulties is attenuated when parents report high levels of coparenting support and high levels of positivity. In particular, parent positivity attenuated the association between economic stressors and mothers’ mental health whereas coparenting support mitigated the association between economic stressors and fathers’ mental health. Our findings are consistent with a large body of research showing that certain family characteristics operate as buffers or protective factors at particular levels (Davis et al., 2018, 2020), but go beyond it by pointing to more targeted approach to intervening with mothers and fathers and suggest two distinct pointes of intervention. The importance of the coparenting relationship for fathers’ mental health and optimism for mothers’ mental health cannot be emphasized enough. We know that parents who are less anxious and depressed tend to be more positive parents, which is important for children’s wellbeing (e.g., Catalino et al., 2014). Investment in both mothers’ and fathers’ mental health should be a priority for programs. Our findings present a coherent narrative that supporting and investing in parents’ mental health, not just to relieve depression and stress but also to strengthen being hopeful and positive about the future and supporting the coparent relationship are significant mechanisms that can promote wellbeing and protect families against the negative effects of adversity and challenges.

### Limitations

The study is of course not without limitations. First, it is difficult to reveal the directions of associations with a cross-sectional design. Although moderation effects were tested, longitudinal studies are essential for better understanding of the underlying mechanisms between pandemic-related risks and familial functioning. Second, not all items from the original scales were included for several variables. The number of items included in the questionnaire were shorten to decrease the burden on our respondents, who were already very stressed by the pandemic. Therefore, it is not possible to compare these outcomes with the norms established by these scales. Although the items selected for this study have overall adequate content coverage, this could compromise the validity of measures with fewer items because the items that are deleted may contain content that’s important to the concept one is measuring. Third, because of time, logistics, and limited funds we were not able to directly assess children. Although our socioemotional measures are summed across both parents when both parents responded, thus somewhat reducing the measurement error, using parents’ report of children’s behaviors is not ideal. Last but not least, our models account for relatively small amount of variance in the child outcome measures. About 14% of child socioemotional problems and 18% of child prosocial behaviors are accounted for.

### Conclusion

In summary, these results make clear that the consequences of the economic and social pandemic-related stressors on family functioning are still revealing themselves and are similar but also different for mothers and fathers. In a relatively short period of time, the pandemic has drastically and dramatically altered many aspects of our lives, including children’s, in ways that have yet to be known. Understanding how mothers and fathers use their resources, including psychological resources, to protect themselves and their families is now more important than ever, as the economic and social cost of the pandemic may be the most damaging and enduring that we have experienced in a generation.

## Data Availability Statement

The summary data supporting the conclusions of this article will be made available by the authors, without undue reservation.

## Ethics Statement

The studies involving human participants were reviewed and approved by the Office for Human Research Protections, University of Maryland, College Park and the Committee for Protection of Human Subjects at the University of California, Irvine. The patients/participants provided their written informed consent to participate in this study.

## Author Contributions

NC and SR conceived and designed the study. MH, JR, TC, AA, AM, and MK collected the data and coded the data. MH and JR performed the statistical analyses. NC and MH contributed to the analyses. TC, AA, AM, and MK conducted the literature review, and contributed equally. MH and NC wrote the manuscript, SR, TC, AA, and AM provided feedback/writing of some sections, and all authors provided feedback. All authors edited and gave final approval for publication and were accountable for this work.

## Funding

Research reported in this publication was supported by the Eunice Kennedy Shriver National Institute of Child Health & Human Development of the National Institutes of Health under Award Number R01HD078547 to NC and SR. The content is solely the responsibility of the authors and does not necessarily represent the official views of the National Institutes of Health. This work was also completed with the sponsorship from the University of Maryland President’s Postdoc Fellowship awarded to MH, the first author.

## Conflict of Interest

The authors declare that the research was conducted in the absence of any commercial or financial relationships that could be construed as a potential conflict of interest.

## Publisher’s Note

All claims expressed in this article are solely those of the authors and do not necessarily represent those of their affiliated organizations, or those of the publisher, the editors and the reviewers. Any product that may be evaluated in this article, or claim that may be made by its manufacturer, is not guaranteed or endorsed by the publisher.

## Footnotes

^1^ https://www.qualtrics.com

^2^RStudio Team (2020). RStudio: Integrated Development for R. RStudio, PBC, Boston, MA URL http://www.rstudio.com
